# Young children (6–7 years) can meaningfully participate in cognitive interviews assessing comprehensibility in health-related quality of life domains: a qualitative study

**DOI:** 10.1007/s11136-025-03940-z

**Published:** 2025-03-05

**Authors:** Victoria Gale, Philip A. Powell, Jill Carlton

**Affiliations:** School of Medicine and Population Health, University of Sheffield, Sheffield, UK

**Keywords:** Patient reported outcome measures, Cognitive interview, Young children, Comprehensibility, Content validity

## Abstract

**Purpose:**

Establishing the comprehensibility of patient reported outcome measures (PROMs) in quality of life research is essential. Cognitive interviews are recommended as a ‘gold standard’ for evaluating comprehensibility among adult populations but are not routinely used with young children (≤ 7 years). The current study therefore aimed to evaluate the feasibility of cognitive interviewing using traditional and adapted methods with children aged 6–7 years to evaluate PROM item comprehensibility.

**Methods:**

Fourteen children (6–7 years) with a range of diagnosed health conditions participated in individual cognitive interviews. Each child answered six mock PROM items (physical, psychological, and social health-related quality of life domains) and concurrent verbal probes were used to evaluate item comprehensibility. Interviews were audio recorded and transcribed verbatim. Transcripts were analysed using a novel Comprehensibility Continuum which coded the extent of alignment between children’s explanations of items and intended meanings.

**Results:**

Cognitive interviews were successful; extent of comprehensibility could be determined for 83/84 (99%) item discussions. Most items were comprehensible, with children describing the intended item meaning for 74/84 (88%) items evidenced by contextual examples and/or de-contextual definitions in children’s responses to verbal probes. Three items (‘walk’, ‘sad’, and ‘made fun of’) were identified as requiring further testing and/or refinement, where a lower percentage of discussions contained evidence of intended item meaning.

**Conclusion:**

Despite previous uncertainty, this study demonstrates how methodological challenges can be addressed to enable young children’s participation in cognitive interviews evaluating item comprehensibility, ultimately contributing to the accurate measurement of young children’s health outcomes in healthcare and research.

**Supplementary Information:**

The online version contains supplementary material available at 10.1007/s11136-025-03940-z.

## Introduction

Systematic measurement of children’s health-related quality of life (HRQoL) and related constructs via patient reported outcome measures (PROMs) is essential for incorporating children’s own views and experiences into healthcare, research and policy [1–4]. Children should be enabled to self-report via PROMs wherever possible [2, 5] and the development of children’s PROMs has received increasing attention over time [4, 6, 7]. During development and evaluation processes, content validity of PROMs needs to be demonstrated [5, 8]. Instruments should accurately capture constructs of health that are meaningful to the intended target population [5, 8–10] and should be relevant, comprehensive, and comprehensible [11].

The need to establish content validity, particularly comprehensibility, is magnified when developing self-report PROMs for young children (i.e., ≤ 7 years) [12]; still-developing cognitive and linguistic skills can affect how young children understand and complete PROMs [12, 13]. Crucially, it cannot be assumed that young children will interpret PROM content consistently with *adult* developers [14, 15]. To account for childhood development, guidance recommends establishing instrument content validity in narrow age bands for *all* ages the PROM is intended for (i.e., it cannot be assumed what is comprehensible for an 11-year-old will also be comprehensible for a 6-year-old) [2, 5, 13].

Cognitive interviews (or cognitive ‘debriefing’ interviews) are recommended for establishing comprehensibility (as well as other aspects of content validity) during instrument development/evaluation for both adults [5, 9, 16, 17] and children [2]. Typically, target population members complete the PROM while simultaneously saying all their thoughts out loud (“think aloud”) and/or being asked direct questions (“verbal probes”) to establish their thinking during PROM completion [16–18]. The International Society for Pharmacoeconomics Research (ISPOR) Good Practices Task Force recommends using the information to evaluate and address problems with instrument comprehensiveness and comprehensibility (e.g., of items, response options, recall period, instructions, etc.) [17]. ISPOR provides a set of guidelines for cognitive interview design and implementation with adults [17] but not with children, despite potential methodological challenges associated with including children in qualitative PROM development often being recognised [2, 13].

The abstract nature of cognitive interviews raises concerns that they may be too challenging for young children [13, 19]; they are considered challenging even for adults [20, 21]. Young children can struggle with abstract, hypothetical questions and may struggle to verbally explain their thinking or elaborate on how they responded to PROM items [13]. Further, they may not fully understand their role as ‘evaluators’, instead focussing on completing the PROM [2], and may not have long-enough attention spans to evaluate an entire instrument [3, 13, 22, 23].

Identifying ways to address these challenges can be difficult; unlike the ISPOR guidance for adults [17] there are no standard guidelines for cognitive interviewing with young children, and general recommendations for qualitative PROM development with children primarily focus on older children and adolescents [2, 13, 19]. Additionally, a recent review found very few published examples of children’s (4–7 years) involvement in cognitive interviews as part of PROM development/evaluation, most of which contained little reporting detail explaining *how* young children were enabled to participate [24]. It is unclear whether young children’s limited involvement is because they are incapable of participating, or because methods of interviewing have not been suitably adapted to enable their participation [24].

In summary, the feasibility of cognitive interviewing with young children to support evaluation of PROM comprehensibility is unknown [24], potentially impacting how content validity can be established for PROMs developed for this age group. This study aimed to establish the feasibility of cognitive interviewing with children aged 6–7 years for comprehensibility evaluations. Reporting follows the Standards for Reporting Qualitative Research (SRQR) [25] (Online Resource (OR) 1: Supplement 1).

## Methods

### Design

Cognitive interviews were conducted with children aged 6–7 years. “Feasibility” was operationalised as it being possible to gather meaningful data from children that could be used to identify the extent of alignment between children’s interpretations of items and intended item meanings (i.e., support comprehensibility evaluations). Interviews were reparative; they aimed to identify and repair problems with comprehensibility [21], as is typical within cognitive interviewing for PROM development/evaluation [17].

### Recruitment and participants

Eligible participants were children aged 6–7 years with diagnosed health conditions (of any severity) who could engage in a speaking activity with an adult in English. Children with health conditions were recruited to ensure the sample reflected typical participant characteristics in cognitive interview studies. Targeted recruitment was conducted in collaboration with three UK primary schools (based in Sheffield and Walsall). Together the interviewer (VG) and school staff identified potentially eligible children and shared information about the project with parents/guardians. Consent was obtained from parents/guardians on behalf of their child, but parents/guardians were encouraged to involve their child in decision-making; information stories and videos for children about the research were provided. Target sample size was 14 (seven each aged 6- and 7-years), as per the COnsensus-based Standards for the selection of health Measurement INstruments (COSMIN) [16]. Seven-year-olds were recruited and interviewed before recruitment began with 6-year-olds.

### Setting

Interviews took place in the child’s school (e.g., in the school library); school settings were practical for parents/guardians and for the time and resources available for the project. Parents/guardians could be present for the interview if they and/or their child wished. They were instructed to not answer questions on behalf of their child, but reasonable support was allowed if their child looked to them for help (e.g., repeating a probe, gentle redirection to the interview task).

### Mock PROM items

To retain focus on exploring feasibility of cognitive interviewing rather than evaluating existing instruments, 12 mock PROM items were developed to be evaluated in interviews. Mock items were intended to reflect typical items found in commonly used generic children’s PROMs (OR 1: Supplement 2). Items covered physical, psychological, and social HRQoL domains, used a recall period of “today”, and each had three response levels (Table 1) (OR 1: Supplement 2). Input from Patient & Public Involvement (PPI) groups of parents and teachers of young children (OR 1: Supplement 2) confirmed items were likely understandable for children aged ~ 7 years.

**Table 1:** Mock patient-reported outcome measure (PROM) items evaluated in cognitive interviews

Health domain	Item	Intended meaning	Essential nature	Definitive attributes
Physical	Poorly 1. I do not feel poorly today 2. I feel a bit poorly today 3. I feel really poorly today	Feeling unwell, sick, under the weather, not very well. Symptoms like headache, stomach ache, temperature etc	Feeling unwell/not right	Physical symptoms
	Walk 1. I cannot walk today 2. I can walk a bit today 3. I can walk a lot today	Walk, go somewhere, use legs	To be able to move using legs	To go somewhere
	Pain 1. I am not in pain today 2. I am in a bit of pain today 3. I am in a lot of pain today	Physical hurt, a part of your body hurts	Physical hurt	A part of your body hurts
	Sleepy 1. I am not sleepy today 2. I am a bit sleepy today 3. I am really sleepy today	Feeling tired, wanting to go to sleep, haven’t had enough sleep	Feeling tired/lacking energy	Needing more sleep/wanting to go to sleep
Psychological	Sad 1. I am not sad today 2. I am a bit sad today 3. I am really sad today	Upset, down, low, something might have made you feel sad	Feeling upset/low	Not being okay, something made you sad
	Angry 1. I am not angry today 2. I am a bit angry today 3. I am really angry today	Feeling mad or cross about something, it might make you want to shout or cry	Feeling mad/cross about something	Physical manifestations
	Worried 1. I am not worried today 2. I am a bit worried today 3. I am really worried today	Feeling nervous, a bit scared something might happen	Feeling nervous or on edge	Anxious about something that might happen/you are unsure of
	Scared 1. I am not scared today 2. I am a bit scared today 3. I am really scared today	Frightened of something or that something might happen, made to ‘jump’	Frightened/worried about something	Can be made to ‘jump’
Social	Get on well with 1. I cannot get on well with friends today 2. I can get on well with friends a bit today 3. I can get on well with friends a lot today	Can join in with friends, able to talk, not falling out or arguing or making each other upset, being happy with each other	Can join in with others positively	Can play and talk without arguing/falling out/making each other upset
	Lonely 1. I am not lonely today 2. I am a bit lonely today 3. I am really lonely today	Feel alone, isolated, no friends, nobody to play with or talk to	Feel alone/isolated	Nobody to talk to, play with, or be friends with
	Made fun of 1. Other children have not made fun of me today 2. Other children have made fun of me a bit today 3. Other children have made fun of me a lot today	Other children have been mean or laughed at me	Other children have been mean/laughed at you	Other children have been unkind
	Join in with 1. I cannot join in with friends today 2. I can join in with friends a bit today 3. I can join in with friends a lot today	Play with others, can ‘do’ the game and talk	To be able to play with others	You can talk and ‘do’ the game with others

**Fig. 1:**
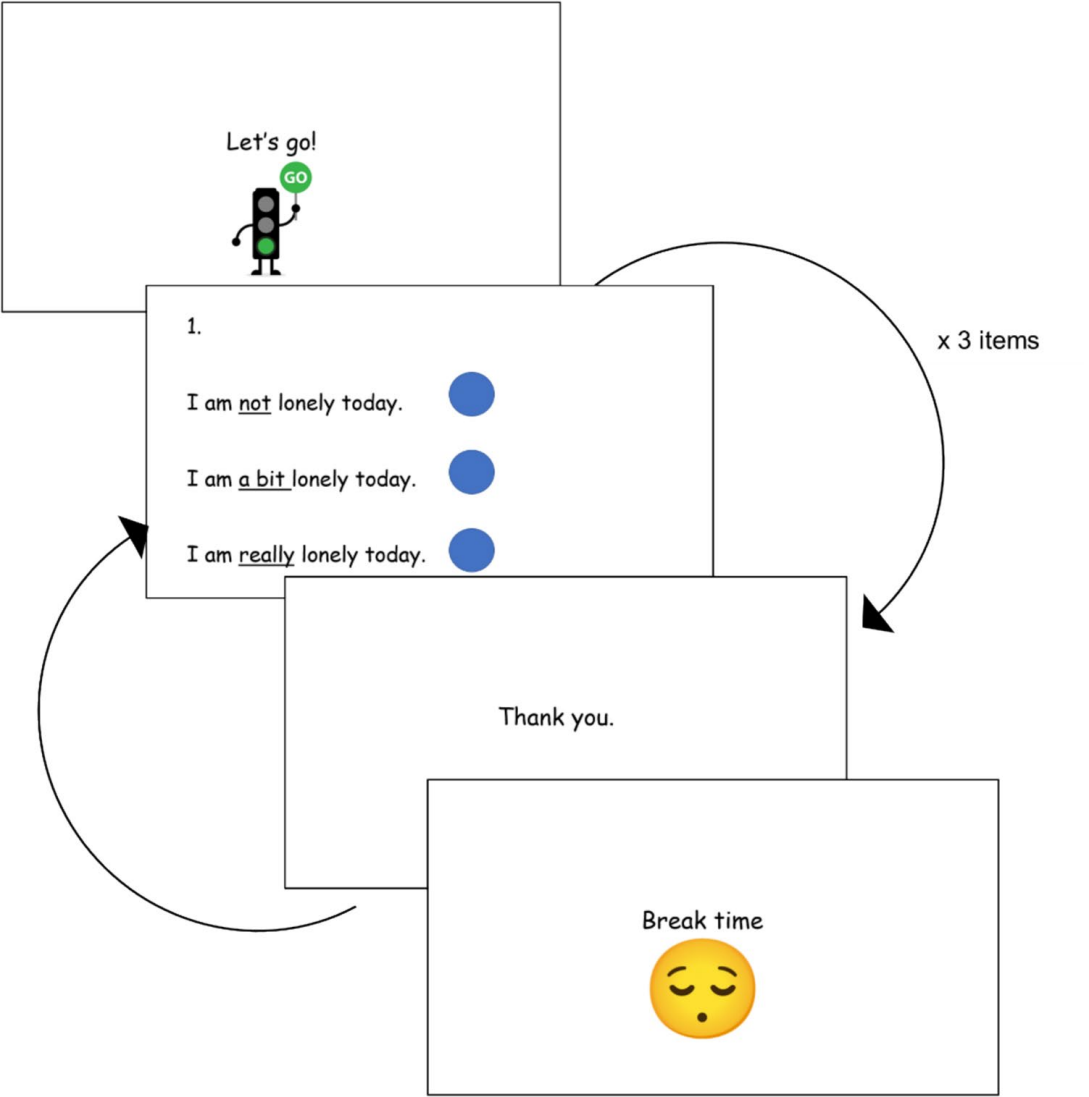
Example presentation of mock patient reported outcome measure items

Mock items were presented individually on a touch screen laptop (Fig. 1) and children were instructed to select the response option that was “most like them”. All items were read out loud by the interviewer. Each child completed six mock items with presentation order randomised. Items were presented in two blocks of three with a break between blocks (although children could take additional breaks if requested) (Fig. 1).

### Interview methods and procedure

Interview methods followed the same general recommendations as for adults [17] but with several modifications to be appropriate for young children informed by recommendations for qualitative PROM development [2, 13, 19] and broader health research with children [22, 23, 26–30], PPI input (OR 1: Supplement 2), and an initial pilot study with five children aged 7 years (OR 1: Supplement 3).

#### Introductions and rapport-building

**Fig. 2:**
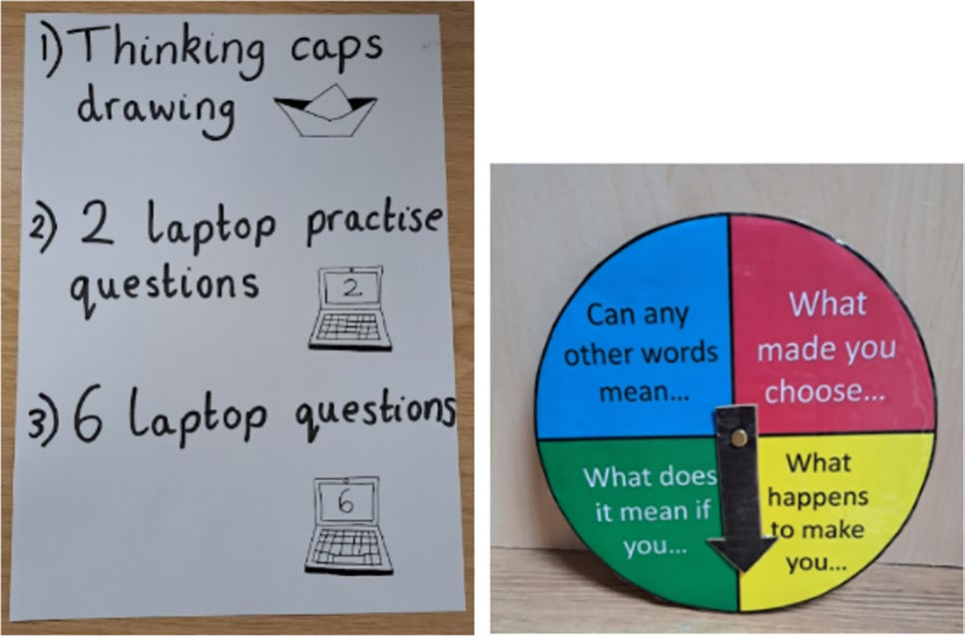
Example visual timetable listing interview activities and spinner prop used to ask follow-up probes

Where possible, the interviewer first spent time with the child/children being interviewed in their lessons and introduced them to the interview space before interviews began [22, 23, 28, 29]. At the start of individual interviews, the research and the child’s ethical rights were explained using visual prompts [22, 23, 29, 30] and audio recording equipment was demonstrated [23, 30]. The interviewer and child made a visual timetable listing interview activities to support children’s understanding of the interview process (Fig. 2). Finally, the child and interviewer completed a creative activity (e.g., drawing) of the child’s choosing to build rapport [22, 23, 28, 29].

#### Cognitive interview

After responding to an item, children were asked multiple verbal probes [21] (the pilot study (OR 1: Supplement 3) found think aloud techniques to be ineffective):

1. “What does [item] mean to you?” (standard probe recommended by ISPOR [17], and recommended for children [2, 13]).
2. “Teach Teddy” paraphrasing activity: ISPOR recommends asking participants to explain what items mean in their own words [17], but paraphrasing is considered too challenging for young children [13]. Research within Developmental Psychology has shown young children can demonstrate higher levels of cognition when tasks are presented concretely with clear purposes [31]. As such, children were shown a toy bear, told he did not understand the item, and asked to help by explaining the item to him – “Can you explain to Teddy what [item] means?”.
3. Follow-up “Spinner” probes. Multiple probes may be needed to fully understand children’s interpretations of items [13]. Four follow-up probes were presented visually on a toy-spinner (Fig. 2), including “Can any other words mean [item]?”, “What made you choose [item]?”, “What does it mean if you feel/are [item]?”, and “What happens to make you feel/think [item]?”. As with “Teach Teddy”, the spinner was intended to concretely represent the interview task by visually demonstrating that the interview involved answering questions about PROM items.

Probes were administered until the interviewer judged enough information had been gathered to inform an evaluation of item comprehensibility according to a decision tree (OR 1: Supplement 4). Typically, probes were administered in the order listed above, but this was adapted if children either responded well to, or struggled with, a particular probe. Interview guides were not amended throughout the course of the interviews. Two practise items not related to health (“I *don’t* like sweets/I like sweets *a bit/a lot*” and “I *don’t* like running/I like running *a bit/a lot*”) were first presented to enable children to practise responding to multiple-choice questions and answering the verbal probes. Next, the children were presented with and discussed the mock PROM items individually. Children were offered a certificate and stickers as thanks for their participation.

Interview sessions were limited to a maximum of 60 minutes, inclusive of study explanations and rapport-building activities conducted one-to-one. Data collection took place between June 2023 and March 2024. Interviews were conducted by VG, a PhD student who was trained in cognitive interview methods and had previous experience working as a primary school teacher. Interviews were audio recorded [16, 17] and written records were made of non-verbal cues and contextual information relevant to analysis.

### Analysis

Analysis aimed to evaluate item comprehensibility i.e., the extent to which children’s interpretations of PROM items aligned with intended item meanings. If it was possible to evaluate comprehensibility from collected data, it would suggest that cognitive interviewing with young children was feasible.^1^ Current guidelines for, and approaches to, cognitive interview analysis do not clearly detail how comprehensibility analysis should be conducted. As such, to enhance the trustworthiness and credibility of analysis, a novel approach was developed (described in full elsewhere [32]) – the Comprehensibility Continuum (CC) – that operationalises ‘alignment’ between intended and interpreted item meaning and enables systematic identification of evidence for comprehensibility from cognitive interview data by multiple researchers [32]. The CC was developed based on a pre-existing continuum used to measure young children’s semantic word knowledge [33].

Prior to analysis, intended item meanings were agreed by the research team, defined according to their “essential nature” (i.e., fundamental meaning) and “definitive attributes” (i.e., primary characteristics) (Table 1). To ensure data accuracy, the interviewer (VG) transcribed all audio recordings verbatim. Transcription involved the removal of all potentially identifiable information and the inclusion of relevant contextual notes made throughout the conduct of the interviews. Transcripts were coded deductively in iterative sets using the CC (Table 2); CC codes described the extent to which children’s explanations of items aligned with intended meanings, including “(0) No relevant response”, “(1) No/incorrect knowledge”, “(2) Schematically-related knowledge”, “(3) Contextual knowledge”, “(4) De-contextual knowledge”, and “(5) Paired knowledge”. Definitions and examples of codes are included in Table 2. All codes ≥ Level 3 indicate the participant captured the essential nature of the intended item meaning in their explanation (i.e., the item was understood as intended).

**Table 2:** Comprehensibility Continuum (CC) used to code cognitive interviews (adapted from [32])

	Code		Definition	Example(s)
Response does not capture essential nature of intended meaning	0	No relevant response	Participant does not provide a response that can be used to evaluate item comprehensibility	No response
				Off-topic response
	1	No/incorrect knowledge	Response is not aligned with intended item meaning	Incorrect knowledge – response is completely unrelated to intended meaning
				Not known – participant says they do not know
				Meaning of a similar sounding word – participant describes a word that sounds similar to the target item
	2	Schematically-related knowledge	Response misses the essential nature/definitive attributes of the intended item meaning but does demonstrate partial understanding. There is no clear distinction between knowledge of target item and schematically related words	Overextension or under extension – participant’s explanation extends or restricts the intended item meaning e.g., *“It means when you can’t do anything”* (overextension for ‘sleepy’)
				Meaning of a structurally related word i.e., target word + prefix e.g., *“You have a bad head”* (describing ‘headache’ rather than ‘ache’)
				Connotation – participant describes an idea or feeling that the target item invokes
				Non-definitive attributes – response contains related, but not definitive, attributes of the target item e.g., “*When you go to bed*” (relates to potentially feeling ‘tired’ but does not capture essential nature of needing sleep/rest)
				Dummy subordinate – response includes repetition of the target item with a dummy subordinate i.e., a word that only serves a grammatical function such as “it” in the sentence “It’s getting late”. E.g., *“Something aches”* (‘something’ is the dummy subordinate’)
				Identified by opposite – response describes the opposite of the target item e.g., *“awake”* instead of “tired”
Response does capture essential nature of intended meaning	3	Contextual knowledge	The essential nature of the intended item meaning is captured in an example/meaningful context. The response must contain at least one idea that is referred to by a specific noun or verb e.g., *“Ache means when your legs are hurting all night, like when they are growing”*	
	4	De-contextual knowledge	The essential nature of the intended item meaning is described in a way that is not couched in a contextual example. The response must show evidence of generalisation of the intended meaning such as by a formal definition or synonym. E.g., *“A part of your body might ache if it is painful or hurts for a long time”*	
	5	Paired knowledge	The response combines both contextual and de-contextual knowledge e.g., *“Ache means when you have something that hurts for a long time, like growing pains in your legs at night”*	

Initially codes were assigned to all responses given to verbal probes in item discussions. Next, the highest code within each item discussion was assigned as the overall CC code for the child’s interpretation of the item [32]. If relevant to evaluating comprehensibility, additional notes were made alongside codes (e.g., if the child demonstrated both understanding that aligned with intended meaning but also provided an alternative interpretation of the item, such as “pain” meaning emotional hurt as well as physical hurt). All authors were involved in coding. VG coded all 14 transcripts and JC and PP independently dual coded four transcripts in two iterative sets [32]. After each set, all researchers met to discuss coding decisions and reach consensus over disagreements. VG then reviewed and updated coding for non-dual coded transcripts to reflect key learnings from the dual coding discussion [32]. Clear audit trials were maintained to document coding decisions. A formal inter-coder reliability score was not calculated – in-keeping with the CC method, emphasis was instead placed on discussion between coders to promote reflection and development of a systematic approach to coding [32].

Overall CC codes were analysed descriptively to determine the extent to which children provided data sufficient for comprehensibility analysis; overall codes ≥ Level 1 would indicate responses were sufficient (i.e., they would contain evidence that the child either did or did not capture the intended item meaning in their explanation) while overall codes of Level 0 indicate that the child had not provided a response relevant to comprehensibility analysis. Overall CC codes were tabulated and compared across participants to identify each item’s comprehensibility according to a pre-defined comprehensibility threshold; ≥ 85% of overall CC codes for an item need to be ≥ Level 3 for an item to broadly be considered comprehensible [32].

## Results

### Sample and interview characteristics

Fourteen interviews were conducted, seven each with children aged 7 years and 6 years (Table 3). Each child responded to all six presented mock PROM items, resulting in a total of 84 item discussions. Participating children had a range of diagnosed health conditions including different allergies (e.g., asthma, eczema, and food allergies), gastrointestinal, heart, hormone, and physical joint conditions, developmental delay, and neurodiverse conditions (including Autism and Attention Deficit Hyperactivity Disorder (ADHD)). Parents were present for three interviews and school staff were present for four interviews. Total length of interviews (including rapport-building, breaks, and the cognitive interview segment) ranged from 15 to 60 minutes. Cognitive interview segments ranged from 9 to 21 minutes.

**Table 3:** Sample characteristics

Characteristics	N (%)
Gender	
Male	5 (35.7%)
Female	9 (64.3%)
Age	
6 years	7 (50%)
7 years	7 (50%)
School year group*	
1	1 (7.1%)
2	11 (78.6%)
3	2 (14.3%)
Ethnic identity	
White British	10 (71.4%)
Asian/Asian-British	3 (21.4%)
Mixed/Multiple ethnic groups	1 (7.1%)

*Children were recruited from school year groups 1 (aged 5–6 years), 2 (ages 6–7 years) and 3 (ages 7–8 years). Only children aged 7 years were eligible to take part and this was confirmed during recruitment and with the child at the start of the interview

### Overall CC codes

**Fig. 3:**
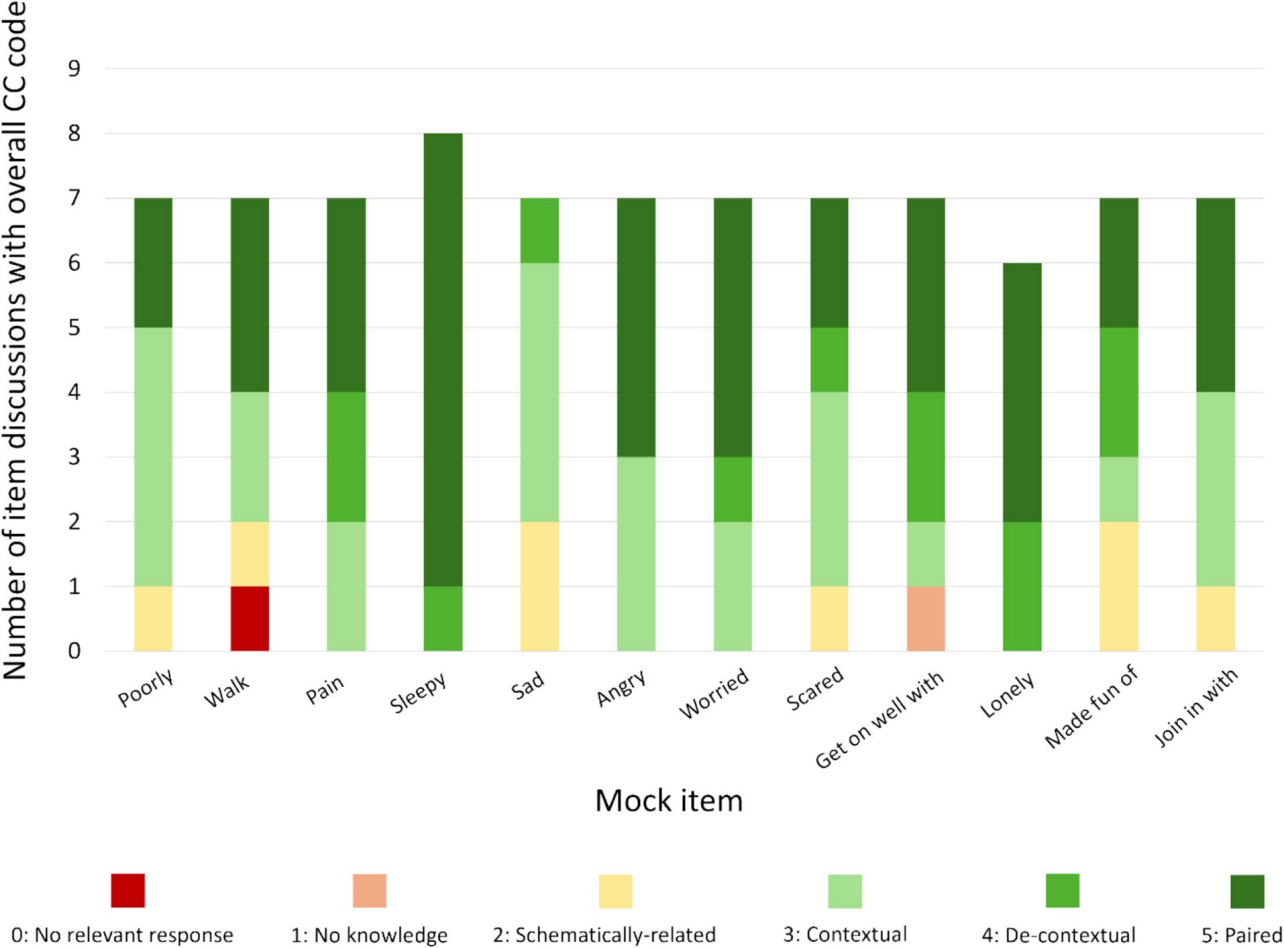
Bar chart showing overall Comprehensibility Continuum (CC) codes for each mock PROM item

Figure 3 shows the overall CC codes for each item discussion (n = 84) (all anonymised data is available in OR 2). Only one item discussion was assigned an overall CC code of “0 – No relevant response” (for the item “walk”—Fig. 3). All other discussions had overall CC codes that indicated the child had provided a response that was relevant to comprehensibility evaluation i.e., ≥ Level 1. Most overall CC codes (74/84 (88%)) were ≥ Level 3 indicating that children articulated the essential nature of the intended item meaning in the majority of item discussions. Further, children often provided substantial detail in their responses by including both contextual and de-contextual examples and definitions – Level 5, which requires both contextual and de-contextual explanations, was the most common overall CC code, assigned to 37/84 (44%) item discussions. For example, one 7-year-old described the meaning of “sleepy” as:

*“Sleepy means when you are a bit tired. I’m a bit tired because I didn’t get any sleep today – my cat kept on waking me up!”* (P05. Coded as “(5)” because the response contained a de-contextual explanation of “sleepy” that included a synonym – “tired” – and a contextual example of feeling sleepy; a lack of sleep because they were woken up).

Similarly, a 6-year-old described the meaning of “get on well with friends” as:

*“It means that you’ve had a great time with them and you’ve had lots of fun and you’ve smiled to your friends and they’ve smiled back to you […] my friends was letting me play. They was letting me join in with what they was doing”* (P11. Coded as “(5)” because the response contained both a de-contextual explanation and a specific contextual example).

Further example participant quotes for each item are included in Table 4. Overall CC codes were comparable across ages; 38/42 (90%) and 36/42 (86%) overall codes were ≥ Level 3 for 6- and 7-year-olds respectively. Codes were also comparable across item domains; overall codes ≥ Level 3 were assigned to 26/29 (90%) physical item discussions, 25/28 (89%) psychological item discussions, and 23/27 (85%) social item discussions. There was some variation between participants in overall CC codes with P05, P08 and P14 having more overall CC codes < Level 3 compared to other participants (Table 5).

**Table 4:** Example participant quotes for each item (all anonymised data is available in Online Resource 2)

Item	Participant quote	Participant (age)	Comprehensibility Continuum code assigned to participant quote
Poorly	*“It’s not good”*	P13 (6 years)	2 – schematically-related
	*“you’re just like sitting but you can’t do any like homework or you can’t do any colouring and you feel like very tired”*	P06 (7 years)	3 – contextual
	*“Feeling poorly means that you don’t feel well and that you feel like you’re going to puke […] once I puked in the school toilets”*	P11 (6 years)	5 – paired
Walk	*“it means when like your leg is very bad […] before I couldn’t walk because my leg was very bad and I had to have like a test to see how my ankle is […] there was a bone that cracked”*	P05 (7 years)	3 – contextual
	*“It means that you’re moving your legs [swinging alternate legs under the table to demonstrate] […] so you can move and your whole body can move”*	P14 (6 years)	5 – paired
Pain	*“Pain means if your hip’s hurting […] or your belly”*	P02 (7 years)	3 – contextual
	*“It means that you’ve hurt yourself”*	P09 (6 years)	4 – de-contextual
	*“Pain means that you have hurt yourself really badly or weight goes into something or the most painful thing I can imagine […] is a tarantula bite if you’re allergic to it it hurts”*	P12 (6 years)	5 – paired
Sleepy	*“Erm feeling sleepy is that you’re erm really tired”*	P01 (7 years)	4 – de-contextual
	*“I woke up really early […] it means that you don’t have that much energy in you […] tired”*	P11 (6 years)	5—paired
Sad	*“So if you feel sad its like upset and water coming out of your eyes and really upset”*	P08 (7 years)	4 – de-contextual
	*“If you’re sad it means someone hit you or they hurt your feelings. Like I’m a bit sad because [name] said she won’t be my friend”*	P02 (7 years)	3 – contextual
Angry	*“You might like to shout. You might chuck something”*	P13 (6 years)	3—contextual
	*“Angry means when you’re mad and when you hit people it’s when you been naughty, and angry means when you can’t control your anger, sometimes that happens to me sometimes, and […] angry means when you want to fight someone”*	P05 (7 years)	5 – paired
Worried	*[the dog] “had to go to the vets”*	P04 (7 years)	3 – contextual
	*“You might feel a little bit scared”*	P13 (6 years)	4 – de-contextual
	*“It means that you’re a bit sad and a bit frightened. But most the thing that makes me worried is somebody beating me up”*	P09 (6 years)	5 – paired
Scared	*“It means you don’t like something and it makes you have tears”*	P01 (7 years)	2 – schematically related
	*“It means if there’s a ghost or spider or if there’s a moth and if you’re scared of sunlight!”*	P10 (6 years)	3 – contextual
	*“In case I get told off by my teacher and I don’t like being told off. And in case I do something wrong. So it always happens to us these feelings and feeling scared means you’re like afraid of something or you’re afraid if something’s going to happen”*	P12 (6 years)	5 – paired
Get on well with friends	*“[name] is like taking all my friends away and then he’s making everyone else play with him”*	P03 (7 years)	3 – contextual
	*“That means when you have friends and you can play very well with them and they’re not making you upset. Playing very well is when you can hug and give each other a big hug and hold hands and play games with each other”*	P08 (7 years)	5—paired
Lonely	*“You’re a bit left alone”*	P09 (6 years)	4 – de-contextual
	*“It means that you ain’t got no friends to play with and you feel a bit sad and then once I didn’t have no friends so I had to play and after they met up with me when I was like three and then we just becomed friends again and we’re still friends in the same class”*	P07 (6 years)	5 – paired
Made fun of	*“It means that them [not] being kind. It would not be okay if they keep on not saying good things to you just tell the teacher”*	P01 (7 years)	4 – de-contextual
	*“It means they are being mean to a person, and they’re making fun of your skin colour, your gender, and what your eye colour is”*	P11 (6 years)	5 – paired
Join in with friends	*“Because like I asked [name] to join in to play and they said yeah”*	P02 (7 years)	3 – contextual
	*“I can join in my friends a lot today coz I played with them at break time and lunch time”*	P06 (7 years)	3 – contextual
	*“Yeah because most of people that I only play with are out there [name] and [name] my friend that chases me […] it means that you’re playing with someone”*	P09 (6 years)	5—paired

**Table 5:**
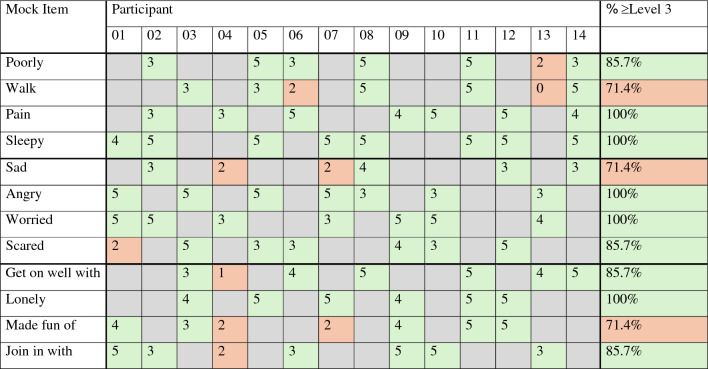
Comparison of overall Comprehensibility Continuum (CC) ratings across participants

### Elicitation of essential nature of item meaning

**Fig. 4:**
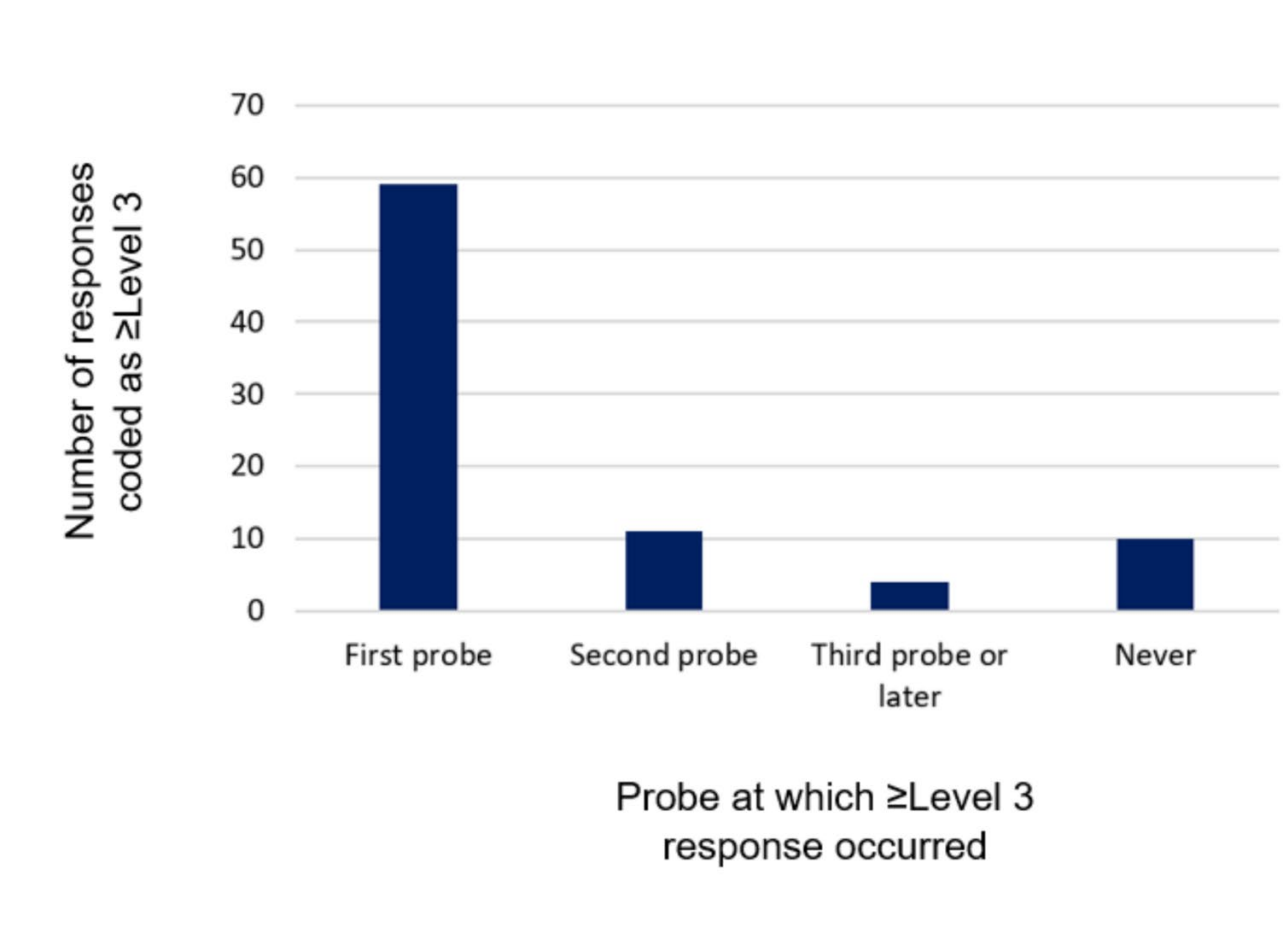
Bar chart showing the number of responses coded as ≥ Level 3 after the first, second, and third or later probe in discussion of an item

A total of 74/84 (88%) item discussions were coded overall as ≥ Level 3. Within these discussions, children’s responses to the first probe mostly captured the essential nature of the intended meaning (59/74 (80%) item discussions) (Fig. 4). This indicates that children were generally able to explain the essential nature of the intended item meaning at the start of item discussions; multiple probes were not needed to demonstrate that they had understood the item as intended.

### Comprehensibility analysis

Three of the mock PROM items did not meet the pre-determined comprehensibility threshold (≥ 85% of item discussions assigned overall codes of ≥ Level 3 to be considered comprehensible) – “walk”, “sad”, and “made fun of” (Table 5). If the purpose of this study was to develop/refine a real PROM, these items would likely need to be evaluated further in additional cognitive interviews. For each of these items, two item discussions were assigned an overall CC code less than Level 3 (Table 5):

#### Mock item: “walk”

One item discussion was coded as “(0)” because the child did not respond to any verbal probes; they were shy at the beginning of the interview when “walk” was discussed, potentially accounting for their lack of response. The other item discussion was coded as “(2)” because the responses did not capture essential nature (using legs to physically move somewhere) but did contain non-definitive attributes (having strong legs):

*“It means that like your legs are like very strong and healthy and you do exercise a lot”* (P06).

#### Mock item: “sad”

Both item discussions were coded as “(2)” – responses did not capture the essential nature of being upset. One child described only non-definitive attributes of feeling sad (i.e., crying):

*“It means you have water coming down here [points to eyes and cheeks]”* (P07).

The other identified “sad” by its opposite:

*“I am not happy”* (P04).

#### Mock item: “made fun of”

Both item discussions were coded as “(2)” – responses did not capture the essential nature of another child having been unkind. One child described an affective connotation by stating that it was “*difficult*” to play with other children if they make fun of you, and the other described affective connotations (feeling sad and worried) and non-definitive attributes (telling a teacher):

“*You might be a bit sad and you might be a bit worried and you might want to go home and you might have to tell the teacher*” (P07).

## Discussion

This study demonstrates new evidence for the feasibility of cognitive interviewing to evaluate PROM item comprehensibility with children aged 6–7 years. Evidence for alignment between intended and interpreted item meaning was clearly operationalised and identified in children’s responses to verbal probes; data was sufficient for evaluating item comprehensibility and for identifying items requiring further testing and/or refinement. Furthermore, children’s responses were often highly detailed, illustrating their understanding of items through both de-contextual and contextual explanations, and where interpretations aligned with intended meaning, children did not require extensive probing.

This study addresses gaps in previous literature and directly challenges the view that children ≤ 7 years cannot participate in cognitive interviews or will only provide limited data. Guidance for involving young children is scarce and there exists only a small number of published examples of children ≤ 7 years having been involved in cognitive interviews as part of PROM development [24]. Suggestions of the youngest age from which children can participate in cognitive interviews vary from potentially 5 years [2], to definitive statements that children younger than 8 cannot self-report via questionnaires so would not be involved in cognitive interviews [34]. It has also been proposed that children aged 6–8 years likely cannot elaborate on their thoughts and will only respond with ‘yes/no’ answers in cognitive interviews [13]. This study provides novel feasibility evidence that children aged 6–7 years old can provide comprehensibility data in PROM development and evaluation.

By implementing the Comprehensibility Continuum (CC), this study is the first of its kind to clearly report how evidence for alignment between intended and interpreted item meaning was defined. Previous studies with young children have not elaborated on how evidence for item comprehensibility was identified, as is typical within current approaches to cognitive interview analysis [32]. For example, Tomlinson et al. rated 4–7-year-olds’ interpretations of items from “completely incorrect” to “completely correct” but did not describe how judgements were made regarding the ‘correctness’ of the child’s interpretation [35]. Using rigorous analysis methods in this study further demonstrates the feasibility of cognitive interviewing with 6–7-year-olds.

The findings of this study have significance when considering the development/evaluation of children’s PROMs globally. To be considered meaningful measures in clinical trials, regulatory bodies, such as the Food and Drug Administration (FDA), require evidence of PROM content validity via qualitative development processes, including cognitive interviews, in narrow age bands [5, 8, 17]. In demonstrating the feasibility of gathering meaningful comprehensibility data, this study also supports the development of self-report instruments as are preferred over proxy-reports by the FDA when measuring non-observable states (e.g., pain intensity) [2, 5]. Findings also have implications for PROM implementation in clinical practice and healthcare systems where concerns regarding comprehensibility have been identified as a barrier to routine implementation of children’s instruments [36].

This study clearly describes how cognitive interview techniques were implemented to enable useful data to be gathered from young children. Although necessary for the development of best practices [27, 37], comprehensive reporting of cognitive interview methods used with young children is limited in previous studies [24]. There is concern that young children’s attention spans are too short for cognitive interviewing [13]; here it is demonstrated that 6–7-year-olds could discuss six items in depth for up to 20 minutes, which could help the design of future studies. Although using multiple probes may increase confidence in understanding children’s interpretations of items, the current study found extensive probing was not necessary for children to explain interpretations, contrary to some existing suggestions [13]. The adapted techniques (i.e., ‘Teach Teddy’) may be useful for future studies; data elicited from these techniques was comparable to data elicited from the standard ISPOR probe. In particular, using physical props may be advantageous in situations where young children need support maintaining attention on the interview task [13, 28, 30].

This study is not without limitations. Only item comprehensibility was investigated but multiple PROM components (e.g., response items, recall period, instructions) and aspects of content validity (e.g., comprehensiveness, relevance) should also be evaluated in cognitive interviews during PROM development/evaluation [16, 17]. Verbal probing techniques used in the current study could potentially be used to evaluate comprehensibility of other PROM components, and the principle of adapting interview tasks to be concrete could be applied to evaluating comprehensiveness and relevance. Further testing is required to establish the feasibility of cognitive interviewing with young children to evaluate *all* PROM components and aspects of content validity, and to confirm the applicability of findings to the development/evaluation of ‘real’ PROMs.

Only children aged 6–7 years participated in the study, meaning the feasibility of including children from 4- and 5-years remains unclear. However, chronological age does not equal ability [12, 13], and results were comparable across 6- and 7-year-olds. It is therefore possible that children aged 4–5 years can also participate in cognitive interviews, warranting further research. This study was conducted with a sample of children from the UK where formal schooling begins earlier (usually at 4-years-old) than is typical in most countries [38]. The transferability of findings to other countries, and to other languages, thus requires further testing. It is possible that type of health condition may have influenced a child’s engagement with the interview. As an initial study exploring the feasibility of cognitive interviewing with a small sample size, it was not considered appropriate or practical to formally explore potential effect of health condition on interview engagement; this would require further research. However, the quality and usefulness of children’s responses to verbal probes was consistent across the sample, with all except one item discussion providing the information necessary for comprehensibility evaluation. These initial findings are encouraging in suggesting young children with a range of health conditions can, in principle, participate in cognitive interviews.

## Conclusion

Cognitive interviewing to evaluate PROM item comprehensibility was feasible with children aged 6–7 years. Clear evidence for feasibility was demonstrated via the novel Comprehensibility Continuum. Although including younger age groups in cognitive interviews may require different methodological considerations, interview methods used in this study were not vastly different from those typically used with older children and adults. PROM developers therefore have a responsibility to include young children in cognitive interviews wherever possible, ultimately to ensure the systematic and accurate measurement of children’s health outcomes.

## Supplementary Information

Below is the link to the electronic supplementary material.

Supplementary file1 (PDF 887 KB)

Supplementary file2 (XLSX 83 KB)

## Footnotes

1 Feasibility was not dependent on developing a set of PROM items that were all comprehensible; rather it would be demonstrated by it being *possible* to evaluate *extent* of item comprehensibility.
