# Supplementary material for: Young children (6–7 years) can meaningfully participate in cognitive interviews assessing comprehensibility in health-related quality of life domains: a qualitative study
Source: Qual Life Res. 2025 Mar 5;34(6):1633–46. doi: 10.1007/s11136-025-03940-z (PMC12119656; doi:10.1007/s11136-025-03940-z)
Supplement: Supplementary file 1 — Supplementary file1 (PDF 887 KB) [file 11136_2025_3940_MOESM1_ESM.pdf]

Online Resource 1: Supplements 1-4

Article Title: Young Children (6-7 years) can Meaningfully Participate in Cognitive Interviews Assessing Comprehensibility in Health-Related Quality of Life Domains: A Qualitative Study

Journal: Quality of Life Research

Authors: Victoria Gale[a], Philip A. Powell[a], and Jill Carlton[a]

Affiliation: [a]School of Medicine and Population Health, University of Sheffield, UK

Corresponding author: Victoria Gale; vargale1@sheffield.ac.uk

Table of Contents

Supplement 1: Standards for Reporting Qualitative Research (SRQR) checklist 2

Supplement 2: Patient and Public Involvement and Mock PROM item Development 5

1 Patient and Public Involvement 5

2 Development of mock PROM items 8

Supplement 3: Pilot Study 11

Supplement 4: Verbal Probing Decision Tree 21

Supplement 1: Standards for Reporting Qualitative Research (SRQR) checklist

Standards for Reporting Qualitative Research (SRQR)*

http://www.equator-network.org/reporting-guidelines/srqr/

Title and abstract

	Page/line no(s).
**Title** - Concise description of the nature and topic of the study Identifying the study as qualitative or indicating the approach (e.g., ethnography, grounded theory) or data collection methods (e.g., interview, focus group) is recommended	Title
**Abstract** - Summary of key elements of the study using the abstract format of the intended publication; typically includes background, purpose, methods, results, and conclusions	Abstract

Introduction

**Problem formulation** - Description and significance of the problem/phenomenon studied; review of relevant theory and empirical work; problem statement	Introduction
**Purpose or research question** - Purpose of the study and specific objectives or questions	Introduction (paragraph 6)

Methods

**Qualitative approach and research paradigm** - Qualitative approach (e.g., ethnography, grounded theory, case study, phenomenology, narrative research) and guiding theory if appropriate; identifying the research paradigm (e.g., postpositivist, constructivist/ interpretivist) is also recommended; rationale**	Methods - 'Design'
**Researcher characteristics and reflexivity** - Researchers' characteristics that may influence the research, including personal attributes, qualifications/experience, relationship with participants, assumptions, and/or presuppositions; potential or actual interaction between researchers' characteristics and the research questions, approach, methods, results, and/or transferability	Methods - 'Interview methods and procedure'
**Context** - Setting/site and salient contextual factors; rationale**	Methods - 'Setting'
**Sampling strategy** - How and why research participants, documents, or events were selected; criteria for deciding when no further sampling was necessary (e.g., sampling saturation); rationale**	Methods - 'Recruitment & Participants'
**Ethical issues pertaining to human subjects** - Documentation of approval by an appropriate ethics review board and participant consent, or explanation for lack thereof; other confidentiality and data security issues	Methods - 'Recruitment and Participants'<br><br>Statements and Declarations - 'Ethics

	Approval', 'Consent to Participate', 'Consent to Publish'
**Data collection methods** - Types of data collected; details of data collection procedures including (as appropriate) start and stop dates of data collection and analysis, iterative process, triangulation of sources/methods, and modification of procedures in response to evolving study findings; rationale**	Methods - 'Interview methods and procedure'
**Data collection instruments and technologies** - Description of instruments (e.g., interview guides, questionnaires) and devices (e.g., audio recorders) used for data collection; if/how the instrument(s) changed over the course of the study	Methods - 'Interview methods and procedure'
**Units of study** - Number and relevant characteristics of participants, documents, or events included in the study; level of participation (could be reported in results)	Results - 'Sample and Interview characteristics'
**Data processing** - Methods for processing data prior to and during analysis, including transcription, data entry, data management and security, verification of data integrity, data coding, and anonymization/de-identification of excerpts	Methods - 'Analysis'
**Data analysis** - Process by which inferences, themes, etc., were identified and developed, including the researchers involved in data analysis; usually references a specific paradigm or approach; rationale**	Methods - 'Analysis'
**Techniques to enhance trustworthiness** - Techniques to enhance trustworthiness and credibility of data analysis (e.g., member checking, audit trail, triangulation); rationale**	Methods - 'Analysis'

Results/findings

**Synthesis and interpretation** - Main findings (e.g., interpretations, inferences, and themes); might include development of a theory or model, or integration with prior research or theory	Results
**Links to empirical data** - Evidence (e.g., quotes, field notes, text excerpts, photographs) to substantiate analytic findings	Results (throughout) and Table 4

Discussion

**Integration with prior work, implications, transferability, and contribution(s) to the field -** Short summary of main findings; explanation of how findings and conclusions connect to, support, elaborate on, or challenge conclusions of earlier scholarship; discussion of scope of application/generalizability; identification of unique contribution(s) to scholarship in a discipline or field	Discussion
**Limitations** - Trustworthiness and limitations of findings	Discussion

Other

**Conflicts of interest** - Potential sources of influence or perceived influence on study conduct and conclusions; how these were managed	Yes - under sub-headings at end of manuscript

**Funding** - Sources of funding and other support; role of funders in data collection, interpretation, and reporting	Yes - under sub-headings at end of manuscript

*The authors created the SRQR by searching the literature to identify guidelines, reporting standards, and critical appraisal criteria for qualitative research; reviewing the reference lists of retrieved sources; and contacting experts to gain feedback. The SRQR aims to improve the transparency of all aspects of qualitative research by providing clear standards for reporting qualitative research.

**The rationale should briefly discuss the justification for choosing that theory, approach, method, or technique rather than other options available, the assumptions and limitations implicit in those choices, and how those choices influence study conclusions and transferability. As appropriate, the rationale for several items might be discussed together.

Reference:

O'Brien BC, Harris IB, Beckman TJ, Reed DA, Cook DA. **Standards for reporting qualitative research: a synthesis of recommendations.** *Academic Medicine*, Vol. 89, No. 9 / Sept 2014 DOI: 10.1097/ACM.0000000000000388

Supplement 2: Patient and Public Involvement and Mock PROM item Development

This supplement includes details regarding the Patient and Public Involvement (PPI) activities conducted to inform the development of the project. It also describes the process through which mock PROM items were developed for evaluation in cognitive interviews.

1 Patient and Public Involvement

Patient and Public Involvement (PPI) is the involvement of members of the public in the research process to conduct research 'with' as opposed to 'on' them [1]. Ideally, this should take place throughout the research process, including research design, implementation, and dissemination, as the intention is to ensure research is being conducted that the people who it is intended for need and value. Given the time and resource constraints of the PhD project it was not possible to implement PPI in full. This is a limitation of the work. However, PPI it was incorporated into the development of the research methods through two groups recruited from a UK primary school (located in Bristol).

1. **Parent/family group** – the aim of consulting this group was to provide input on participant recruitment and to identify ways of approaching parents about the project such that they would be interested in their children taking part. This would also involve feedback on consent forms and information sheets.
2. **Teacher group** – the aim of consulting this group was to provide input on how the research could be implemented practically, including the best times of year/day for children in schools to take part in research (e.g., not in the weeks before Christmas, not in mornings when children are typically taught core subjects), and feedback on potential methods (e.g., phrasing of questions such that they are likely to be understood, recommendations for toys/activities to use or avoid).

In-person discussion groups were held in November 2022. Example summaries of feedback and proposed actions from these groups are detailed in Table 1 below

Table 1. Example summaries of feedback and action points from PPI groups

Topic	Feedback summary	Proposed actions	Outcomes and implementation
Inviting parents and children to take part in the research	While emails are quick, extra forms of communication may be helpful to 'stand out' from other regular emails e.g., sharing the research in school newsletters, reminder texts, physical information sheets/consent forms<br><br>Provide clear information about the purpose and expected impact of the project, exactly what children will be asked to do (including examples), how data will be protected and managed securely, and how much time parents and children will need to commit<br><br>If possible, come into schools to share information, or provide a video – show parents that I am a real person!	1. Discuss with alternative ways of communicating with parents and families with schools taking part in the project, including visiting in-person<br>2. If possible, share draft information sheets/videos and consent forms with focus groups for further feedback	1. During discussions with collaborating schools, the option for me to visit in-person during recruitment was always given.<br>2. Pilot information sheets were shared with parent PPI group and feedback was incorporated
Asking parents about their child's health to inform sampling	The existing rating scale is too general and unlikely to produce useful information. Be clear about whether I need to know about children's medical conditions, or their health and wellbeing in general. Clearly define what is meant by these. One alternative could be to ask "Do you consider your child to have a medical condition, either physical or mental? Yes/No" with the option to disclose what this is	1. Discuss with supervisors what information we need about children's health and adjust accordingly (being clear about what 'health' is in the question)	1. It was decided that purposive sampling would aim to recruit children with ***diagnosed*** medical conditions to address any ambiguity
General feedback on the practicalities	Ensure that the interviews take place in a 'child-friendly' environment e.g., range of toys, rooms they are familiar with	1. Discuss with participating schools to find a suitable environment and to have appropriate resources and toys available	1. During discussions with collaborating schools suitable locations and resources for the interviews were requested. I also took multiple books and toys to the

of the interviews	Be introduced as a ‘safe’ adult before starting the interviews to support rapport with children and help them feel comfortable. A video explaining the task will be helpful<br><br>In general, ensure that the tasks on the iPad are not too wordy and have visuals to support what is being said	2. Create a video for children explaining what they will be asked to do in the interview and what their role as the ‘expert’ is. This would be shared with parents and class teachers to discuss with the children taking part	interviews in case the school could not provide these on the day.<br>2. Videos were created for both children and parents and used during recruitment

2 Development of mock PROM items

Mock PROM items were developed to cover three broad domains of health as defined by the World Health Organisation (WHO) [2] – physical, psychological, and social – and to align with commonly used generic health-related quality of life (HRQoL) instruments that include self-report options for children aged ≤7 years (Table 2).

Table 2. Commonly used generic paediatric PROMs measuring HRQoL that enable children aged ≤7 years to self-report

Instrument name	Age from which children can self-report
Child Health Utility – 9 Dimension (CHU-9D) [3]	7 years
EQ-5D-Y [4]	5 years
Pediatric Quality of Life Inventory 4.0 (PedsQL 4.0) [5]	5 years
KiddyKINDL [6]	4 years

Item words (e.g., tired, unhappy) were extracted from the instruments and categorised according to health domains (Table 3). Synonyms for each of the item words were identified and combined with the item words in a list of candidate items. These were shared with PPI groups to confirm which were typically likely to be understood by children aged 7 years. The final selection of mock items (included in Table 1 in the main text) was decided by the research team and approved by PPI groups.

Table 3. Example item words and synonyms identified from commonly used generic instruments for children aged 4-7 years

Health domain	Sub-domain	Item words from existing instruments	Synonyms
Physical	Physical symptoms	Pain, tired	Poorly, sick, sleepy, hurt
	Physical functioning	Walking, dressing	Move, take care of, put on clothes, play
Psychological	Emotions	Worried, unhappy, sad, annoyed, scared	Upset, afraid, angry, nervous, frightened
	Cognition	Paying attention at school, forgetful	Thinking, not remember

Social	Social function	Join in with activities, get on with others, tease	Take part, get along, good time, worry, bother, lonely, made fun of
	School	School work, homework	Work, job, know what to do

While some research suggests that children aged ≤7 years should only be given dichotomous response options [7], the generic instruments used to inform the development of mock items for this study ranged from using three to five response levels for children aged 4-7 years, and a recent cognitive interview study found children aged 4-7 years could understand three response levels sufficiently [8]. The mock items used in the current study were therefore developed to have three response levels.

Two main approaches to structuring PROM items with response options were identified from the four generic instruments (Table 1):

1. Item ***and*** response option together (CHU-9D and EQ-5D-Y e.g., “I feel a bit worried today”)
2. Item ***separate*** from response options (PedsQL 4.0 and KiddyKINDL e.g., “During the past week I felt ill – never/sometimes/very often”)

To retain the study’s focus on assessment of comprehensibility of items, not on children’s ability to distinguish between response options, mock items were structured with item and response option presented together. It was also anticipated that structuring items in this way would simplify the task for children – children would be shown three items and asked to choose the one that most closely matched how they felt, as opposed to needing to listen to and understand and item, and then choose from a separate list of response options.

References

1. Haywood, K., et al., Patient and public engagement in health-related quality of life and patient-reported outcomes research: what is important and why should we care? Findings from the first ISOQOL patient engagement symposium. Quality of Life Research, 2015. **24**: p. 1069-1076.

2. Organization, W.H., Basic documents: forty-ninth. World Health Organization: Geneva, Switzerland, 2020.

3. Stevens, K J. Working With Children to Develop Dimensions for a Preference-Based, Generic, Pediatric Health-Related Quality-of-Life Measure. Qualitative Health Research. 2010; vol. 20: 340 - 351

4. Wille, N., Badia, X., Bonsel, G., Burström, K., Cavrini, G., Devlin, N., Egmar, A.C., Greiner, W., Gusi, N., Herdman, M. and Jelsma, J., 2010. Development of the EQ-5D-Y: a child-friendly version of the EQ-5D. Quality of life research, 19(6), pp.875-886.

5. Varni, J.W., Seid, M. and Kurtin, P.S., 2001. PedsQL™ 4.0: Reliability and validity of the Pediatric Quality of Life Inventory™ Version 4.0 Generic Core Scales in healthy and patient populations. Medical care, 39(8), pp.800-812.

6. Ravens-Sieberer, U. and Bullinger, M., 1998. Assessing health-related quality of life in chronically ill children with the German KINDL: first psychometric and content analytical results. Quality of life research, 7, pp.399-407.

7. Coombes, L., et al., Enhancing validity, reliability and participation in self-reported health outcome measurement for children and young people: a systematic review of recall period, response scale format, and administration modality. Quality of Life Research, 2021. **30**: p. 1803-1832.

8. Tomlinson, D., et al., Development of mini-SSPedi for children 4–7 years of age receiving cancer treatments. BMC cancer, 2019. **19**: p. 1-9.

Supplement 3: Pilot Study

This supplement describes the pilot study conducted to inform the development of the project methodology.

1 Aim and Research Questions:

The aim of this pilot study was to investigate the feasibility and practicality of the proposed research project intended to evaluate the viability of cognitive interviewing with children aged ≤7 years. It asked the following two questions:

1. Do the methods of cognitive interviewing produce data of sufficient quality such that they would be appropriate for larger-scale testing? Specifically, of the four techniques of cognitive interviewing (described below), which are most appropriate for larger-scale testing and what refinements might be needed?
2. Is the proposed project feasible with regards to collaboration with schools, participant recruitment, time and resources required to conduct and analyse the interviews, and the use of mock PROM items?

2 Methods:

2.1 Sample and recruitment

Participants were children aged 7 years attending a collaborating primary school in Bristol, UK. Information about the project was distributed by the school to all parents/guardians of children aged 7 years. If interested, parents/guardians then completed an online screening survey. Information collected in the survey was used to inform purposive sampling which aimed to recruit six children aged 7 years with a range of demographics and diagnosed health conditions (physical and/or mental and of any severity). Parents/guardians were then contacted with an online consent form. Six children was considered a practical number to recruit given the time and resource limitations of the pilot study.

2.2 Ethics

The pilot study was reviewed and ethically approved by the Division of Population Health Research Ethics Committee at the University of Sheffield (ref. 051410).

2.3 Interview procedure

All interviews took place in the children's school. Parents/guardians could be present in the interview if they or their child chose but were asked to not answer questions on behalf of their child. Interviews were limited to a maximum of 60 minutes, including time to build rapport and to take breaks.

Before and start of interviews –

Effort was made to reduce power imbalances and build rapport between the adult interviewer and the participating children before and at the start of the interviews [1-3]. During recruitment, a video and illustrated information story were shared for children to help explain the purpose of the research to them. The information emphasised to children that it was up to *them* and their parents/guardians to decide whether or not to take part. Interviews took place in a child-friendly location (the school library) which the children regularly used for small-group activities; this ensured that the interview environment did not 'belong' to adults [2, 4].

The interviewer was introduced to the children by a familiar adult in the school (e.g., their class teacher) and was reassured that it was 'ok' for them to take part. All interviews then began with an informal conversation between the interviewer and the child [1, 3-5]. Next, the interviewer shared a printed information story to re-introduce the purpose of the research, explain the child's role in their interview, their ethical rights, and that there were no right or wrong answers [2, 4, 6, 7]. Recording equipment was demonstrated to children and they were invited to test it [2, 3]. Specific interview tasks were then explained and written as a visual timetable on a whiteboard. All interviews were audio recorded.

Following initial introductions and explanations, each child was presented with two of four possible techniques of cognitive interviewing (described below).

1. Think Aloud – 'Thinking Caps'

To make the think aloud task [8] more concrete for young children, a 'thinking caps' game was used. The game involved giving children a physical hat (a 'thinking cap') to wear and being told that this would help them say everything they were thinking out loud. Printed information with visuals was used to explain the thinking caps and the think aloud task to children. Next, the children were given a practise task whereby they were asked to think out loud while completing a simple jigsaw (this was first modelled by the interviewer). Children were then presented with the mock PROM items and were prompted to continue using the thinking cap to think aloud while responding to the items.

2. Verbal Probes – 'Thinking Caps'

In this technique, the same 'thinking caps' prop was applied to verbal probing [8]. Using printed information with visuals, the thinking cap is explained to help children answer questions about their thinking. Children were given a practise task of being asked to build a Lego model following printed instructions, and being asked verbal probes such as "how did you know which brick to start with?" to practise using the thinking cap. Next, the mock PROM items were presented, and verbal probes were used to evaluate item comprehensibility e.g., "What does [this item] mean to you? What made you choose [this item]?"

3. Paraphrasing – 'Teach Teddy'

The International Society for Pharmacoeconomics Research (ISPOR) taskforce recommend asking participants to paraphrase items in cognitive interviews to evaluate item comprehensibility [8]. However, it has been suggested that this will be took challenging for children aged ≤7 years [9]. To make the paraphrasing probe more concrete and to have a meaningful context for young children,

children were instead asked to explain to a Teddy bear what the items meant e.g., “How would you explain this to Teddy? What should Teddy think about to help him answer this question?”

4. Verbal Probes – ‘Creative Interview’

Creative activities have been recommended to be a useful strategy for supporting children to explore and verbalise their ideas [2, 5]. Children were invited to choose a mock PROM item from an array of cards placed face-down on the table. The interviewer read the chosen card out loud and asked them what they thought the card meant. Several creative activities (e.g., drawing, painting, collage, Lego, playdough) were offered to children to use to help explain their thoughts. Verbal probes were used throughout to explore children’s understanding of the mock PROM items e.g., “Can you tell me about what you are making? What do you think this card means?”

Follow-up probes could be used with all four techniques to elicit further information or seek clarification if needed e.g., “Can you tell me more about that?”. Children were presented with all 12 mock PROM items (see below) for techniques 1-3; the pilot study intended to identify how many mock items was feasible to present to children. For the fourth technique (“Verbal Probes – Creative Interview”) it would not have been possible to evaluate all 12 items in the given time. Hence the printed cards were used and as many as possible were discussed within the interview time. Having completed two techniques, children were offered stickers and a certificate to thank them for their participation.

2.4 Mock PROM items

As with the larger-scale study presented in the main text, mock PROM items were used to retain focus on exploring the feasibility of cognitive interviewing with young children (the development of items is explained in Online Resource 2). For the pilot study, half the mock items were deliberately ‘damaged’ such that they were anticipated to have comprehensibility problems for at least some participants. Similar techniques have been used in previous studies evaluating the effectiveness of cognitive interview methods [10]. If problems were identified with the ‘damaged’ items, it could be inferred that cognitive interviews were successful. A full list of the mock PROM items used in the pilot study is shown in Table 1. Mock PROM items were presented on a touch screen laptop in the same way as has been described in the main text.

Table 1. Mock PROM items used in the pilot study

Item number	Health domain	Item	Intended ‘damage’ where applicable
1	Physical	I <u>don’t/feel a bit/feel</u> really poorly today	N/A
2		I am <u>not/a bit/a lot</u> ailing today	Unknown/challenging vocabulary
3		I am <u>not/a bit/really</u> sleepy today	N/A

4		I have not felt/felt a bit/felt really unwell today	Deliberately vague, likely a less common way of describing health when 7-years-old
5	Psychological	I am not/a bit/really upset today	N/A
6		I am not/a bit/really angry today	N/A
7		I am not/a bit/really thoughtful today	Deliberately vague/ambiguous – could mean being considerate towards others, being contemplative, thinking things through slowly and not rushing etc.
8		I am not/a bit/really dejected today	Unknown/challenging vocabulary
9	Social	Other children have not/a bit/a lot ridiculed to me today	Unknown/challenging vocabulary
10		I cannot/can do some/can do all tasks today	Deliberately vague/ambiguous – could refer to anything an adult requests, could be a task in a game (e.g., playing 'shop keeper') school work etc.
11		I cannot join in with/can join in a bit/a lot with friends today	N/A
12		I cannot/can do a bit/all of my schoolwork today	N/A

'Damaged' items are shown in red

2.5 Analysis

Audio recordings were transcribed verbatim by VG and anonymised in the process. Analysis aimed to evaluate the practicality and feasibility of the project for larger-scale testing and to evaluate whether the data collected would be sufficient to enable an assessment of the comprehensibility of mock PROM items. Data were coded inductively to identify information relevant for evaluating practicality, feasibility, and usefulness. Codes were collated and synthesised narratively. Text summaries [11, 12] were used to summarise children's interpretations of mock item meanings. Summaries were then tabulated to compare responses across the four different techniques of interviewing. Responses were considered of sufficient quality if it was possible to compare intended item meaning to children's interpretations of item meaning. All analysis was conducted by VG and a reflective journal was kept throughout. Results were shared with the project team (JC and PP) who met to reach consensus on modifications needed for the larger-scale project.

3 Results

3.1 Sample characteristics

Six children aged 7 years were successfully recruited, although it was only possible to recruit one child with a diagnosed medical condition. Sample characteristics are shown in Table 2.

Table 2. Sample characteristics (n = 6)

**Characteristics**	**N (%)**
Male	3 (50%)
Diagnosed medical condition	1 (17%)
Year group:*	
2	2 (33.3%)
3	4 (66.7%)
Ethnic identity:	
White British	3 (50%)
White non-British	1 (17%)
Mixed/multiple ethnic groups	2 (33.3%)

*Children were recruited from school year groups 2 (ages 6-7 years) and 3 (aged 7-8 years). Only children aged 7 years were eligible to take part and this was confirmed via the online screening survey.

3.2 Project practicality and feasibility

Recruitment was partly successful; the target sample size was achieved and a ranger of demographics were represented. However, only one child participated who had a diagnosed health condition. An alternative recruitment strategy will therefore be needed to ensure all children in the larger-scale study have diagnosed health conditions.

Five complete interviews took place; one child did not want to participate on the day of the interview and the interviewer judged it appropriate to end the interview early. Length of interview sessions ranged from 25-60 minutes, with the time spent discussing mock PROM items ranged from 20-38 minutes. Parents were present for three of the six interviews and all understood the importance of not responding to verbal probes on behalf of their child.

All children were able to respond to the mock items and differentiate between the three response levels. For example, when probed as to why they had chosen a certain level instead of another, children could justify their responses:

> Interviewer: *"What does that mean if you're a **bit** sleepy?"*
> P01: *"like errr a small amount of sleepy but you're not fully sleepy"*

P05: *"Well it depends how Teddy feels if he's really really tired he might want to go to bed […] if he's not sleepy he might want to be active […] if he's a bit sleepy he might yawn a lot"*

All mock items were presented and discussed in all except one instance; P01 only discussed four items in their second activity ("Think Aloud – Thinking Caps") before becoming distracted and losing focus on the task.

3.3 Evaluation of the interview process

The introduction story used at the start of the interview to explain the interview purpose and children's ethical rights was engaging and effective for communicating important information to children. Children enjoyed the visual timetable and readily interacted with it (e.g., ticking off activities as we completed them). Children also enjoyed interacting with the audio recording equipment at the start of the interview.

Although drawing was originally intended to be used only in the Creative Interview, it was beneficial for establishing rapport with children at the start of all interviews, particularly those who were initially more shy around the interviewer; decorating a thinking cap was offered as a rapport-building activity in interviews 2-6. Several children explicitly stated that they had enjoyed this part of the process:

"Even if I'm not playing with my friends I'm enjoying this" (P05)

3.4 Evaluation of cognitive interview techniques

1. Think Aloud – 'Thinking Caps'

Overall this activity was repetitive and seemingly tricky for children to engage with. P01 lost focus on this task after the first four items and all children who completed this activity needed many prompts to remember to think out loud. Although children's responses had face validity (e.g., when explaining why he had chosen that he did not feel upset today P02 said *"it's because no one laughed at me"*), many additional verbal probes were needed to elicit enough data to support comprehensibility evaluation. Some children also explicitly stated that the task was difficult:

"Tricky mmmm [pause] I don't know what to say" (P06)

2. Verbal Probes – 'Thinking Caps'

This activity was more engaging compared to the think aloud task and the interviewer was able to allow the child to set the pace of the questioning. Probes could be varied which helped to reduce the repetitiveness of the questioning. All three children who completed this task responded to and discussed all 12 mock items. Responses appeared to be trustworthy and had face validity, for example:

Unwell – "*and I have had a bit of a tummy ache*" (P04)

Angry – "*I am not angry today because no one's made me angry um and nothing that I've had has made me angry […] sometimes I get a bit angry with my maths because it could be a bit hard"* (P05)
Tasks – "*like […] answering questions in my reading or something*" (P01)

However, the use of the thinking cap and the practise task was largely redundant; children appeared to understand the task without needing to complete the Lego building activity and the thinking caps were mostly ignored throughout the mock item discussions.

3. Paraphrasing – 'Teach Teddy'

P02 and P05 took part in this technique and the results were different for each. For P02, the activity was less engaging, and he was more interested in completing the items on the laptop as opposed to explaining what Teddy needed to do to answer the questions. He largely gave the same answer for every item e.g.,

"*He needs to think about if he's angry or not*"; "*He needs to think if he's sleepy or not*"; "*He needs to think about if he's upset or not*" (P02)

This required additional probes to be able to establish what the comprehensibility of the item was to him. Differently, the activity was engaging for P05 who enjoyed explaining what the items meant to Teddy and gave more detailed explanations compared to P02 e.g.,

"*It depends if someone's make him upset […] or if you're having fun you might not be upset […] he could feel about what they said*" (P05)

"*It depends how, if how many friends of yours want to play with you*". (P05)

The interviewer used additional probes with P05 as well to elicit further detail, but this was not as necessary as it was for P02. Both children gave trustworthy responses with face validity.

4. Verbal Probes – 'Creative Interview'

The creative activities were engaging for children who spent a long time drawing and painting (interviews that included the creative tasks ranged from 50-60 minutes). Responses to verbal probes largely had face validity and would support comprehensibility evaluations e.g.,

Unwell - "*feeling sick*" (P06)
Angry - "*they might shake their fists if they're angry*" (P04).

Tasks - *"It means um […] do things […] help out […] like if you're going on holiday you could help unpack things at the hotel […] maybe um help the teacher […] and help out if someone gets hurt" (P04)*

However, the number of items possible to discuss was substantially lower than the verbal probes 'Thinking Caps' task (only two mock items were discussed with P04 and four mock items with P06) with no increase in the quality of data.

3.5 Mock items

All children were able to independently response to the mock PROM items. Results for the 'damaged' items were mixed. It was evident that some of these contained unknown vocabulary, and children were confident to ask what they meant. However, the usefulness of using the 'damaged' items to demonstrate the success/unsuccess of the cognitive interviews was not clear. Instead, a more robust approach to evaluating the feasibility of cognitive interviewing would be needed (as is described in the main text with the development of the Comprehensibility Continuum approach to analysis).

4 Summary of and proposed modifications

The pilot study demonstrated the practicality of the proposed project. Recruitment of 7-year-olds through a school was successful and most children were willing to participate in the cognitive interviews. The school provided an appropriate setting for the conduct of the interviews and the workload (i.e., set-up, conduct, and transcription) of interviews was manageable for the time and resources available. The process of presentation of the mock PROM items was successful. Furthermore, the pilot identified direct verbal probing, including an adapted paraphrasing probe, as being potentially viable techniques for gathering data of sufficient quality to enable comparison of intended item meaning to children's interpretations. Overall, the findings warranted further investigation in the larger scale project. Modifications made to the methods for implementation in the larger-sclare project are summarised in Figure 1.

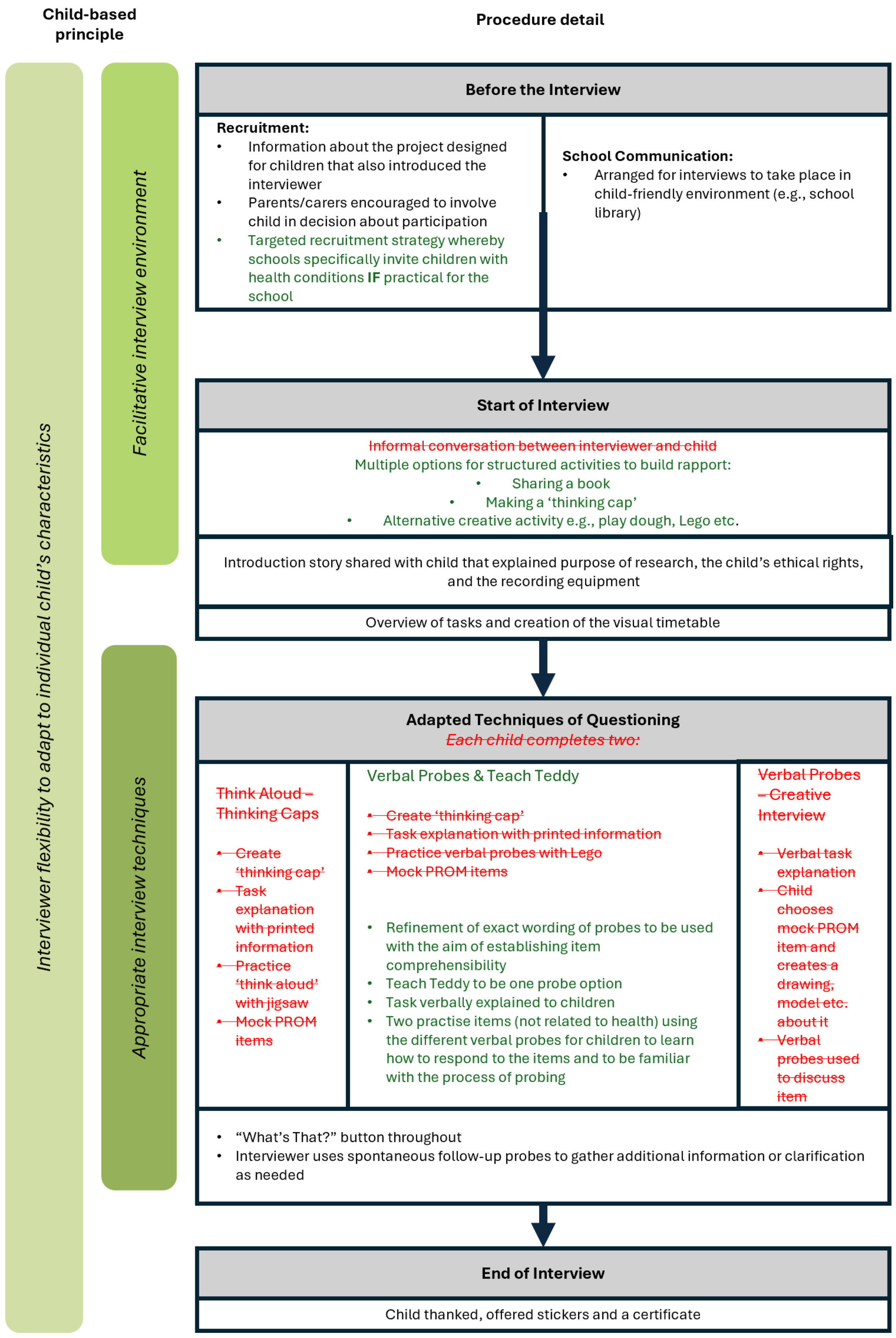

Fig. 1 Proposed modifications for the larger-scale project shown in track changes.

References

1. Kortesluoma, R.L., M. Hentinen, and M. Nikkonen, *Conducting a qualitative child interview: methodological considerations.* Journal of advanced nursing, 2003. **42**(5): p. 434-441.
2. Gill, P., et al., *Conducting qualitative interviews with school children in dental research.* British dental journal, 2008. **204**(7): p. 371-374.
3. Faux, S.A., M. Walsh, and J.A. Deatrick, *Intensive interviewing with children and adolescents.* Western Journal of Nursing Research, 1988. **10**(2): p. 180-194.
4. Curtin, C., *Eliciting children's voices in qualitative research.* The American Journal of Occupational Therapy, 2001. **55**(3): p. 295-302.
5. Huang, X., et al., *Ethical and methodological issues in qualitative health research involving children: A systematic review.* Nursing ethics, 2016. **23**(3): p. 339-356.
6. Patel, Z.S., S.E. Jensen, and J.-S. Lai, *Considerations for conducting qualitative research with pediatric patients for the purpose of PRO development.* Quality of Life Research, 2016. **25**: p. 2193-2199.
7. Matza, L.S., et al., *Pediatric patient-reported outcome instruments for research to support medical product labeling: report of the ISPOR PRO good research practices for the assessment of children and adolescents task force.* Value in Health, 2013. **16**(4): p. 461-479.
8. Patrick, D.L., et al., *Content validity—establishing and reporting the evidence in newly developed patient-reported outcomes (PRO) instruments for medical product evaluation: ISPOR PRO Good Research Practices Task Force report: part 2—assessing respondent understanding.* Value in Health, 2011. **14**(8): p. 978-988.
9. Arbuckle, R. and L. Abetz-Webb, *"Not just little adults": qualitative methods to support the development of pediatric patient-reported outcomes.* The Patient-Patient-Centered Outcomes Research, 2013. **6**(3): p. 143-159.
10. Blair, J. and F.G. Conrad, *Sample size for cognitive interview pretesting.* Public opinion quarterly, 2011. **75**(4): p. 636-658.
11. Willis, G.B., *Analysis of the cognitive interview in questionnaire design*. 2015: Oxford University Press.
12. Knafl, K., et al., *The analysis and interpretation of cognitive interviews for instrument development.* Research in nursing & health, 2007. **30**(2): p. 224-234.

Supplement 4: Verbal Probing Decision Tree

This supplement includes the decision tree used to guide administration of verbal probes in cognitive interviews.

Verbal Probing Decision Tree

Three techniques of probing were used to explore children's interpretations of item meanings:

1. A standard probe recommended by ISPOR [1] – "What does [item] mean?"
2. 'Teach Teddy' paraphrasing activity – "Can you explain what [item] means to Teddy?"
3. Follow-up 'spinner' probes – "Can any other words mean [item]?", "What made you choose [item]?", "What does it mean if you are/feel/have been [item]?", and "What happens to make you feel [item]?"

In addition to these pre-determined probes, the interviewer used both 'wait time' and encouragement where necessary. It is known that young children need longer to process verbal information [2-4] and so the interviewer would wait for at least 10 seconds before moving on to the next probe or item if the child did not initially answer. Similarly, the interviewer would repeat the probe in a slighted altered wording to encourage the child to answer (such as "hmmm I wonder what [item] means to you?") (Table 1) if they did not initially respond or did not respond after 10 seconds.

Table 1. Verbal probes and adjusted wording if wait-time/encouragement is used

**Original verbal probe**	**Wait-encouragement wording**
"What does [item] mean?"	"I wonder what [item] means to you?"
Teach Teddy -<br><br>*"Can you explain what [item] means to Teddy?"*	"I wonder what we could say to explain what this means to Teddy?"
Follow-up spinner probes -<br><br>*"Can any other words mean [item]?", "What made you choose [item]?", "What does it mean if you are/feel/have been [item]?", and "What happens to make you feel [item]?"*	"I wonder if there are any other words that mean…"<br><br>"hmm what made you choose…"<br><br>"I wonder what it means if you…"<br><br>"What might happen to make you feel…"
Generic wait-time/encouragement probes –<br><br>• "hmmm I wonder what [item] means?"<br>• "Oh that was quick! Let's go back – what does [item] mean?"	

A flow diagram (Figure 1) was created to support the interviewer in administering probes consistently across interviews. The interviewer moved on to the next item when either sufficient information was judged to have been gathered to support an evaluation of item comprehensibility, or when the interviewer judged that the child was not willing or able to respond to probes for that particular item. The flow diagram also included instructions for actions to take if a parent intervened in the interview.

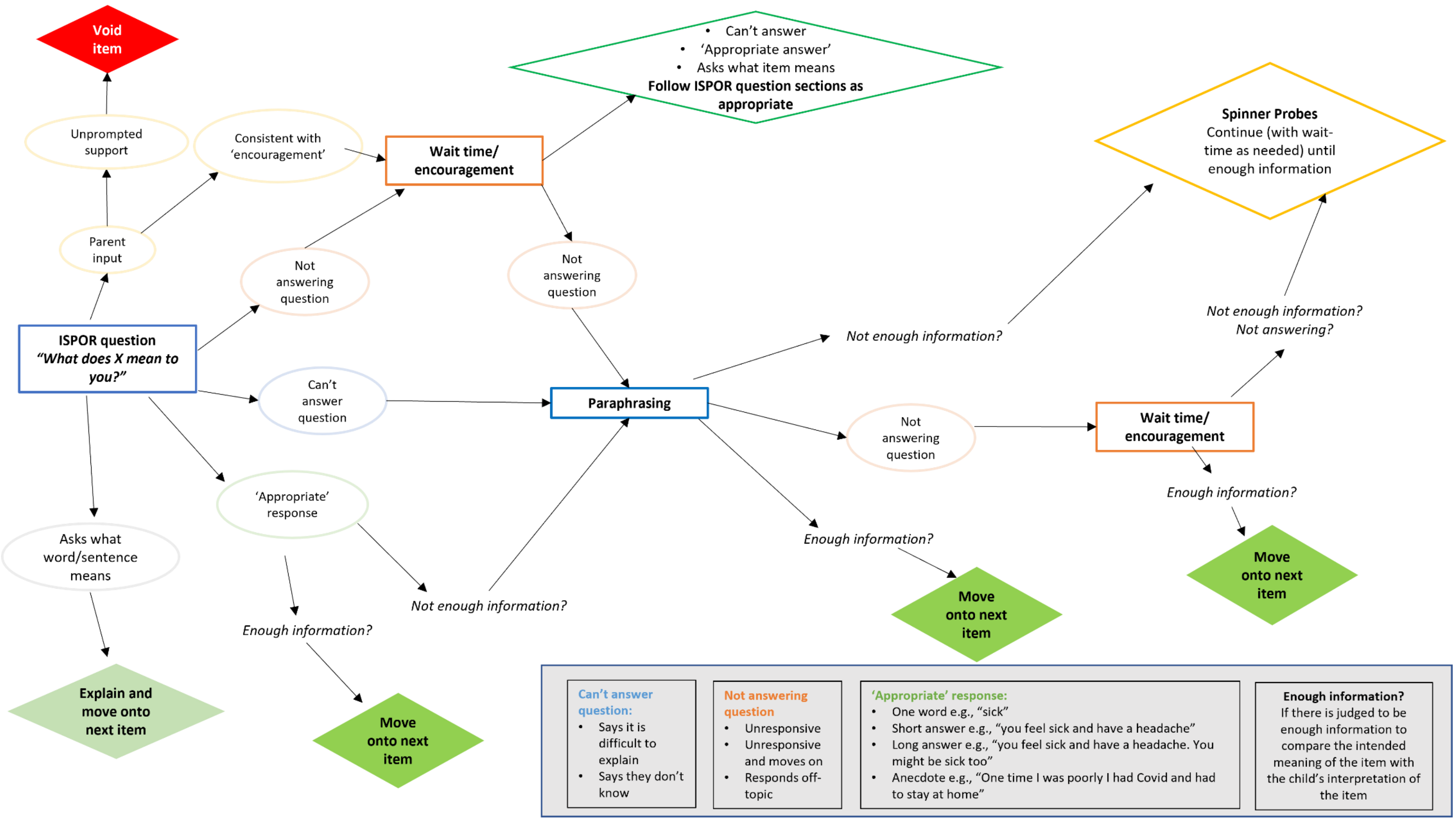

Fig. 1 Decision tree used to support verbal probing in cognitive interviews

References

1. Patrick, D.L., et al., *Content validity—establishing and reporting the evidence in newly developed patient-reported outcomes (PRO) instruments for medical product evaluation: ISPOR PRO Good Research Practices Task Force report: part 2—assessing respondent understanding*. Value in Health, 2011. **14**(8): p. 978-988.
2. Arbuckle, R. and L. Abetz-Webb, *"Not just little adults": qualitative methods to support the development of pediatric patient-reported outcomes*. The Patient-Patient-Centered Outcomes Research, 2013. **6**(3): p. 143-159.
3. Bevans, K.B., et al., *Conceptual and methodological advances in child-reported outcomes measurement.* Expert review of pharmacoeconomics & outcomes research, 2010. **10**(4): p. 385-396.
4. Patel, Z.S., S.E. Jensen, and J.-S. Lai, *Considerations for conducting qualitative research with pediatric patients for the purpose of PRO development.* Quality of Life Research, 2016. **25**: p. 2193-2199.
